# Assessing the Fecal Microbiota: An Optimized Ion Torrent 16S rRNA Gene-Based Analysis Protocol

**DOI:** 10.1371/journal.pone.0068739

**Published:** 2013-07-15

**Authors:** Christian Milani, Arancha Hevia, Elena Foroni, Sabrina Duranti, Francesca Turroni, Gabriele Andrea Lugli, Borja Sanchez, Rebeca Martín, Miguel Gueimonde, Douwe van Sinderen, Abelardo Margolles, Marco Ventura

**Affiliations:** 1 Laboratory of Probiogenomics, Department of Life Sciences, University of Parma, Italy; 2 Departamento de Microbiologia y Bioquimica de Productos Lacteos, IPLA – CSIC, Villaviciosa, Asturias, Spain; 3 Alimentary Pharmabiotic Centre and Department of Microbiology, Bioscience Institute, National University of Ireland, Western Road, Cork, Ireland; 4 INRA, UMR 1319 MICALIS-Microbiologie de l’Alimentation au Service de la Santé humaine, Pôle Ecosystèmes: Interactions des bactéries commensales et probiotiques avec l’hôte, Domaine de Vilvert, Bât 440 R-2 78352, Jouy en Josas, France; University of Glasgow, United Kingdom

## Abstract

Assessing the distribution of 16S rRNA gene sequences within a biological sample represents the current state-of-the-art for determination of human gut microbiota composition. Advances in dissecting the microbial biodiversity of this ecosystem have very much been dependent on the development of novel high-throughput DNA sequencing technologies, like the Ion Torrent. However, the precise representation of this bacterial community may be affected by the protocols used for DNA extraction as well as by the PCR primers employed in the amplification reaction. Here, we describe an optimized protocol for 16S rRNA gene-based profiling of the fecal microbiota.

## Introduction

The gut microbiota is presumed to play a key role in human health and disease through its impact on nutrition, pathogenesis and immunology [1]. During the last decade extensive efforts have been made to determine the complexity of the microbial communities residing in the gut (for review see [2]). In fact, (gut) disorders such as inflammatory bowel disease [3], [4], irritable bowel syndrome [5], obesity [6], [7] and necrotizing enterocolitis [8] have been considered as deviations from a healthy gut microbiota composition.

The capability of high through-put sequencing of 16S rRNA gene sequences by means of Next Generation Sequencing (NGS) technologies has been pivotal in facilitating the discovery of gut microbiota biodiversity [9]. The Ion Torrent PGM instrument represents a recently commercialized bench-top NGS platform and is marketed as being less costly and with a faster turnaround as compared to other NGS techniques such as the 454 and Illumina platforms [10], [11]. Application of the Ion Torrent technology to 16S rRNA-based profiling of complex bacterial communities has been achieved for the investigation of the aquatic microbial community structure of the Athabasca river [12], the bacterial and archaeal community dynamics in a covered anaerobic pond that was utilized to treat waste from a piggery [13], and the microbial population residing in human subgingival plaque [14].

Previous studies have pointed out how the obtained biodiversity image of the gut microbiota is affected by various protocols used for DNA extraction, as well as by the particular PCR primers used for amplification of the targeted region of the 16S rRNA gene [15]–[17], leading to an underestimation of key components of the gut microbiota of infants, in particular bifidobacteria [18]. In fact, based on both culture-based techniques and analysis using species-specific DNA probes, bifidobacteria were considered to represent the dominant component of the neonatal gut microbiota, [19]–[21], though other microbiota studies have suggested that bifidobacteria are present at low abundance or even absent in the infant gut microbiota [22], [23].

These findings reinforce the need for a reliable protocol to investigate the composition of the human gut microbiota. Here, we describe a procedure specifically designed for the Ion Torrent PGM technology to determine the biodiversity of the human gut by means of 16S rRNA gene-based sequence profiling.

## Materials and Methods

### Subject Recruitment and Fecal Sample Collection

The study was approved by the Ethical Committee of the Regional Asturias Public Health Service (SESPA) and informed written consent was obtained from the mothers. All subjects were healthy and had not received any antibiotic or probiotic in the previous 3 months. Stool samples consisted of 6–10 gr of fresh fecal material, and were immediately frozen upon collection at −80°C until processed for DNA extraction.

### Bacterial Strains and Growth Conditions

Ten representatives of abundant microorganisms of the human gastrointestinal tract were used in this study. These include *Bifidobacterium longum* NCIMB 8809, *Collinsella intestinalis* DSM 13280, *Blautia producta* DSM 2950, *Escherichia coli* LMG 2092 and *Klebsiella pneumoniae* CECT 143, which they were grown in de Man-Rogosa-Sharpe (MRS) broth (Difco, Detroit, MI) supplemented with 0.05% (w/v) L-cysteine (Sigma, St. Louis, MO) (MRSC). *Prevotella copri* DSM 18205 and *Blautia coccoides* DSM 935 were cultivated in a combination of Reinforced Clostridial Broth (Merck, Darmstadt, Germany) and Brain-Heart Infusion (Difco), supplemented with 5% (v/v) heat-inactivated fetal bovine serum (LabClinics, Barcelona, Spain). For culturing *Bacteroides thetaiotaomicron* DSMZ 2079, the latter medium was supplemented with 0.005% haemin (Sigma) and 0.005% Vitamin K1 (Sigma). *Faecalibacterium prausnitzii* DSM 17677 was grown in Wilkins-Chalgren Anaerobe broth (Merck), following the recommendations included in the DSMZ medium 339. Finally, an active culture of *Methanobrevibacter smithii* DSM 861, grown in *Methanobacterium* medium (DSMZ 119) was directly supplied by DSMZ.

Cultures were incubated at 37°C in an MG500 anaerobic chamber (Don Whitley Scientific, West Yorkshire, United Kingdom) with an atmosphere of 10% (v/v) H_2_, 10% CO_2_, and 80% N_2_. Taxonomic identity of the microorganisms used for the 16S rRNA gene microbial profiling assays was assessed by sequencing the V1 and V2 variable regions of the 16S rRNA gene using primers plb16 (5′-AGAGTTTGATCCTGGCTCAG-3′) and mlb16 (5′-GGCTGCTGGCACGTAGTTAG-3′) [24].

### PCR Primer Design

Primers Probio_Uni/Probio_Rev (Table 1) were assessed for specificity using the ARB software package [25] and the SILVA 108 SSU Reference 16S rRNA gene database release [26]. In order to validate the designed primers, we used an *in silico* approach based on BLASTN matches with corresponding 16S rRNA gene sequences from various bacteria that are commonly found in the gut as well as those that are not expected in this environment, which provided allowed us to evaluate if these primers may also be used to examine the biodiversity of different microbial ecosystems. In order to increase the number of microbial taxa recognized by the primers designed in this study we introduced a certain level of nucleotide degeneracy.

**Table 1:** Primers used in this study.

Primer name	Adapter sequence	Key	Tag barcode	GAT	Primer Sequence (5′–3′)	Reference
520F	CCATCTCATCCCTGCGTGTCTCCGAC	TCAG	TGAGCGGAAC	GAT	AYTGGGYDTAAAGNG	[28]
802R	CCTCTCTATGGGCAGTCGGTGAT				TACNVGGGTATCTAATCC	[28]
Probio_Uni	CCATCTCATCCCTGCGTGTCTCCGAC	TCAG	TTGGAGTGTC	GAT	CCTACGGGRSGCAGCAG	In this study
Probio_Rev	CCTCTCTATGGGCAGTCGGTGAT				ATTACCGCGGCTGCT	In this study
P1	CCATCTCATCCCTGCGTGTCTCCGAC	TCAG	TCTATTCGTC	GAT	CCTACGGGAGGCAGCAG	[27]
P2	CCTCTCTATGGGCAGTCGGTGAT				ATTACCGCGGCTGCT	[27]
Bact-8F					AGAGTTTGATCCTGGCTCAG	[22], [23]
1391R					GACGGGCGGTGTGTRCA	[22], [23]
Bact-1510R					CGGTTACCTTGTTACGACTT	[32]
ENV1					AGAGTTTGATNNTGGCTCAG	[22], [27], [28], [33]
ENV2					CGGNTACCTTGTTACGACTT	[22], [27], [28], [33]

### Deliberate Contamination of Faeces from Germ-free Rats and DNA Extraction

Pellets for each strain were extensively washed with PBS, concentrated in a PBS solution and cell counts were calculated in a Neubauer Chamber. Feces from germ-free Fisher 344 rats (males) were deliberately contaminated with three different mixes of microorganisms, named mix P1, mix P2 and mix P3, and homogenised for 1 min using a stomacher (IUL Instruments, Barcelona, Spain). In all cases, *M. smithii* was added at a final concentration of 1.30E9 cells/g faeces. In the first mix (P1), both Gram positive and Gram negative microorganisms were added at a final viable count of 3.84 E9 cells/g feces for each strain. In the second mix (P2) Gram positive numbers were the same as in the P1 mix, but Gram negative bacteria were added at a final concentration of 3.84 E7 cells/g faeces for each strain, resulting in a cell population in which the numbers of each Gram negative strain are 2 logs below the numbers of Gram positive strains. Finally, in the third mix (P3) the number of each introduced Gram positive bacterial strain was 2 logs below the Gram negatives: 3.84 E7 cells/g feces for each Gram positive strain, and 3.84 E9 cells/g feces for each Gram negative strain.

Following the homogenization of the various feces-bacterial mixes, four different DNA extraction procedures were used. i) The DNA extraction protocol using the MOBIO Power Soil DNA Isolation Kit (MOBIO Laboratories Inc., Carlsbad, CA) [recommended by the Human Microbiome Consortium (www.hmpdacc.org)] and from here on referred to as ‘MBio DNA-extraction’; ii) The MBio DNA-extraction, which included an initial enzymatic treatment for 1 h (enzymatic mix: Tris 50 mM pH 8.0, 10 mM MgSO_4_, 5 mg/ml lysozyme and 100 U/ml mutanolysin), from here on referred to as ‘MBioEz DNA-extraction’; iii) The QIAamp DNA Stool Mini kit following the manufacturer’s intructions (Qiagen Ltd., Strasse, Germany), from here on referred to as ‘Qia DNA-extraction’; iv) The Qia DNA-extraction protocol including an initial mechanical cell disruption step by inclusion of 0.1 mm zirconium–silica beads (Biospec Products, Bartlesville, OK) and by subjecting the sample to three 1 min pulses at maximum speed in a bead beater (FastPrep FP120 Thermo Savant; Qbiogene, Inc., Illkirch, France) with intervals of 1 min on ice and, subsequently, the mechanical treatment was followed by an enzymatic lysis step for 1 h at 37°C (enzymatic mix: 50 mM Tris-HCl, pH 8.0, 10 mM MgSO_4_, 5 mg/ml lysozyme and 50 U/ml mutanolysin) from here on referred to as ‘QiaEz DNA-extraction’.

### Mouse Trial

All animals used in this study were cared for in compliance with guidelines established by the Italian Ministry of Health. All procedures were approved by the University of Parma, as executed by the Institutional Animal Care and Use Committee (Dipartimento per la Sanità Pubblica Veterinaria, la Nutrizione e la Sicurezza degli Alimenti Direzione Generale della Sanità Animale e del Farmaco Veterinario, Italy). Fecal samples were collected after removal of animals from their box and no sacrifice of the animals was performed.

### 16S rRNA Gene Amplification

Partial 16S rRNA gene sequences were amplified from extracted DNA using primer pair Probio_Uni and/Probio_Rev, which targets the V3 region of the 16S rRNA gene sequence, or employing primer pair P1 and P2 [27], which targets the V3 region of the 16S rRNA gene sequences, or primer pair 520F and 802R [28] corresponding to the V4 region of the 16S rRNA gene sequences. These primers were designed to include at their 5′ end one of the two adaptor sequences used in the Ion Torrent-sequencing library preparation protocol linking a unique Tag barcode of 10 bases to identify different samples. The complete list of the primers used in this study is reported in Table 1.

The PCR conditions used were 5 min at 95°C, 35 cycles of 30 s at 94°C, 30 s at 55°C and 90 s at 72°C, followed by 10 min at 72°C. Amplification was carried out by using a Verity Thermocycler (Applied Biosystems). The integrity of the PCR amplicons was analyzed by electrophoresis on an Experion workstation (BioRad, UK).

### Ion Torrent PGM Sequencing of 16S rRNA Gene-based Amplicons

The PCR products derived from amplification of specific 16S rRNA gene hypervariable regions were purified by electrophoretic separation on an 1.5% agarose gel and the use of a Wizard SV Gen PCR Clean-Up System (Promega), followed by a further purification step involving the Agencourt AMPure XP DNA purification beads (Beckman Coulter Genomics GmbH, Bernried, Germany) in order to remove primer dimers. DNA concentration of the amplified sequence library was estimated through the Experion system (BioRad). From the concentration and the average size of each amplicon library, the amount of DNA fragments per microliter was calculated and libraries for each run were diluted to 3E9 DNA molecules prior to clonal amplification. Emulsion PCR was carried out using the Ion OneTouch™ 200 Template Kit v2 DL (Life Technologies) according to the manufacturer’s instructions. Sequencing of the amplicon libraries was carried out on a 314 chip using the Ion Torrent PGM system and employing the Ion Sequencing 200 kit (Life Technologies) according to the supplier’s instructions. After sequencing, the individual sequence reads were filtered by the PGM software to remove low quality and polyclonal sequences. Sequences matching the PGM 3′ adaptor were also automatically trimmed. All PGM quality-approved, trimmed and filtered data were exported as sff files.

### Sequence-based Microbiota Analysis

The sff files were processed using QIIME [29]. Quality control retained sequences with a length between 150 and 200 bp, mean sequence quality score >25, with truncation of a sequence at the first base if a low quality rolling 10 bp window was found. Presence of homopolymers >6 bp, and sequences with mismatched primers were omitted. In order to calculate downstream diversity measures (alpha and beta diversity indices, Unifrac analysis), 16S rRNA Operational Taxonomic Units (OTUs) were defined at ≥97% sequence homology. All reads were classified to the lowest possible taxonomic rank using QIIME and a reference dataset from the Ribosomal Database Project [30].

OTUs were assigned using uclust [31]. The hierarchical clustering based on population profiles of most common and abundant taxa was performed using UPGMA clustering (Unweighted Pair Group Method with Arithmetic mean, also known as average linkage) on the distance matrix of OTU abundance. This resulted in a Newick formatted tree, which was obtained utilizing the QIIME package.

### Nucleotide Sequence Accession Numbers

The raw sequences reported in this article have been deposited in the NCBI Short Read Archive (SRA) (SAMN02009352, SAMN02009353, SAMN02009354, SAMN02009355, SAMN02009356, SAMN02009357).

## Results and Discussion

### Design of a Suitable PCR Primer Pair for 16S rRNA Gene Sequence Profiling on the Ion Torrent NGS Platform

**Figure 1:**
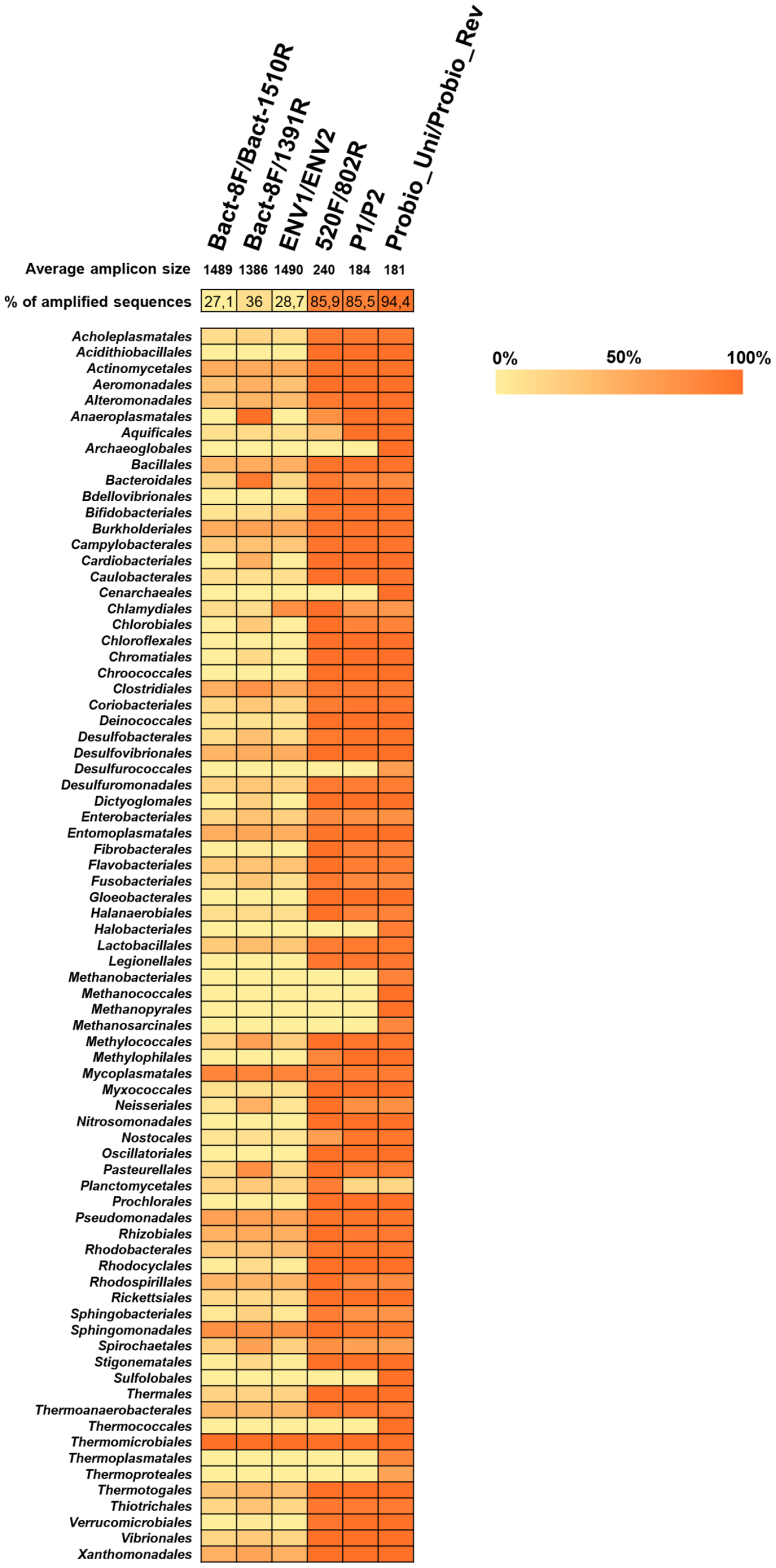
Heat map showing the order classification rates for optimal choice of primers. Cells are colored on a gradient from 0% to 100% matches of the primer with the 16S rRNA gene target sequences of the indicated microbial order. The size of the amplicons generated by the various primer pairs are indicated. Furthermore, the percentage of the amplification rate from each primer pair on the various microbial taxa here described is indicated at the top of the heat map.

Previous investigations on taxonomic classification of the human gut microbiota were based on PCR primers that target the 16S rRNA gene. In order to develop a specific and reliable 16S rRNA gene-based primer set that is suitable for Ion Torrent sequencing technology, we selected, based on currently available literature [27], [28], primer pairs that generate an amplicon of a maximum size of 200 bp and that target the V4 or V3 hypervariable region of the 16S rRNA gene, corresponding to positions 563–797 and 341–534 (coordinates based on the 16S rRNA gene of *Escherichia coli* strain K-12 substr. MG1655). In addition, a novel primer set, i.e. Probio_Uni/Probio_Rev, was designed for the purpose of this study by modification of the well established primer set P1/P2, originally designed by [27]. Compared to other primer pairs mentioned above, the Probio_Uni/Probio_Rev primer pair perfectly matches with a higher number of human gut microbiota components as detected by an *in silico* analysis against Ribosomal Database Project’s (RDP) sequences. As displayed in Figure 1, the Probio_Uni/Probio_Rev primer pair theoretically targets all 16S rRNA gene sequences of the gut microbiota bacterial orders we selected with an average similarity of 94.4% and generating an optimal Ion Torrent amplicon with an average length of 181 bp. Specifically, compared to the primer pair P1/P2 [27], the Probio_Uni/Probio_Rev primer pair perfectly targets (is fully complementary without mismatches) relevant 16S rRNA gene sequences of *Archaeoglobales, Thermococcales, Sulfolobales*, *Cenarchaeales*, *Methanococcales* and *Methanopyrales* (Fig. 1).

### Development of an Appropriate Methodology for DNA Amplification

**Figure 2:**
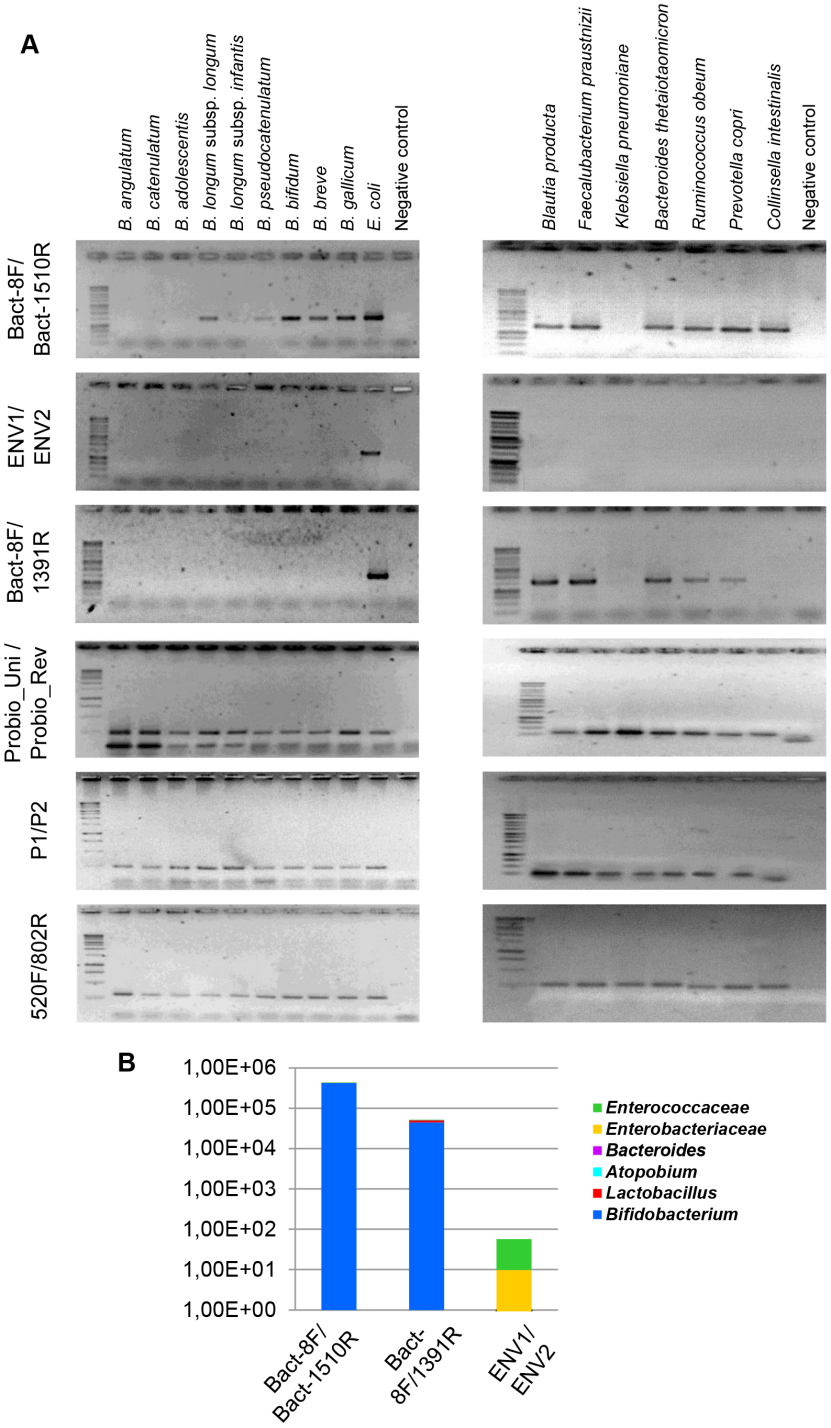
Fecal levels of different microorganisms as analyzed by PCR using different 16S rRNA gene-based primers. Panel a depicts PCR amplification targeting the 16S rRNA gene of different enteric bifidobacteria as well as key intestinal bacteria using the primers described by [22] [27], [32], [33], [43] and the primer set Probio_Uni/Probio_Rev. Panel b. displays the microbial profile of the amplicons generated by the primer sets described by [22], [32], [33] according to the following taxa: *Bifidobacteriaceae*, *Enterobacteriaceae*, *Enterococcaceae*, *Lactobacillales*, *Bacteroidetes* group, and *Atopobium* group.

Precise assessment of the composition of the human gut microbiota crucially depends on the reliability and specificity of the PCR primers employed to amplify the 16S rRNA gene of specific groups of the bacterial community. Comparative assays were performed by amplification reactions using the same samples as templates and either different sets of previously described PCR primers targeting bacterial [32] [22], [27], [28], [32], [33], or the newly designed PCR primer pair (Probio_Uni/Probio_Rev) aimed at amplification of the 16S rRNA gene of key gut microbiota members such as those belonging to the genera *Bifidobacterium*, *Bacteroides*, *Collinsella*, *Prevotella*, *Escherichia*, *Blautia*, *Faecalibacterium*, *Klebsiella* and *Methanobrevibacter*. Notably, no or very little PCR-mediated amplification product was obtained when DNA, extracted from commonly encountered human gut bifidobacterial species (*Bifidobacterium breve*, *Bifidobacterium bifidum*, *Bifidobacterium longum*, *Bifidobacterium adolescentis*, *Bifidobacterium catenulatum*/*Bifidobacterium pseudocatenulatum*, *Bifidobacterium angulatum* and *Bifidobacterium gallicum*) (for review see [34]), was used as a template and employing Bact-8F/Bact-1510R, ENV1/ENV2 and Bact-8F/1319R primer combinations. The dissection by quantitative real-time qPCR of the microbial composition according to several common intestinal microbiota taxa (e.g., *Bifidobacteriaceae*, *Enterobacteriaceae*, *Enterococcaceae*, *Lactobacillales*, *Bacteroidetes* group, and *Atopobium* group) of amplicons obtained from fecal DNA extracted using the method involving an enzymatic lysis based on previously published PCR primers [22], [27], [28], [32], [33] yielded very different microbial profiles (Fig. 2). In particular, the microbial taxon *Bifidobacterium* appears to be completely absent when employing primer combinations ENV1/ENV2 and Bact-8F/Bact-1510R [22], [33]. This is consistent with these primers exhibiting the lowest level of homology with the corresponding 16S rRNA gene sequences of members of the taxa *Bifidobacteriaceae*, *Lactobacillus* and *Enterobacteriaceae* (Fig. 1). Altogether these results lead us to conclude that the apparent lack of bifidobacterial sequences from certain previously published 16S rRNA gene profiling gut microbiota studies is in part due to PCR primers that were biased against key members of the gut microbiota such as bifidobacteria. Thus, many of the published studies describing the bacterial composition of the infant gut seem to have significantly underestimated the incidence and diversity of these commensals.

### Setting up of a Valid Methodology for Sample Processing

Various studies investigating the human fecal microbiota biodiversity have reported highly variable and sometimes contradictory results [22], [23], [35]. Although these results may have been due to biological variation between individuals, it has been shown that sample preparation methods, such as DNA extraction does play a significant role in explaining the reported variations [16]. In order to address this issue we evaluated four fecal DNA extraction methods, i.e. involving different commercial kits and by introducing an initial enzymatic treatment prior to the actual DNA extraction protocol (see materials and methods), and assessed which of these methods would provide the most accurate representation of microbial populations in fecal samples by means of 16S rRNA gene-based profiling using the Ion Torrent PGM technology. Germ-free fecal samples from rats were mixed with various combinations of nine different microbial strains belonging to the following species: *Bifidobacterium longum*, *Collinsella intestinalis*, *Blautia coccoides*, *Blautia producta*, *Faecalibacterium prausnitzii*, *Escherichia coli*, *Prevotella copri*, *Klebsiella pneumoniae*, *Bacteroides thetaiotaomicron* and the Archeal strain *Methanobrevibacter smithii,* representing some of the most abundant bacterial species in the human gastrointestinal tract, according to several reports [36], [37], while *M. smithii* is the most frequently found archeal bacterium in the human gut [38]. Total DNA samples, extracted by different methods from feces contaminated with microbial mixes of these ten microorganisms in different proportions, were used as template for amplification with the primers developed in this study, i.e., Probio_Uni and Probio_Rev. Gnotobiotic feces were used as negative controls in the four extraction procedures, yielding as expected no amplicons. The Ion Torrent sequencing platform, as optimized here, was shown to detect all bacterial strains present in the samples, independent of the relative amount of each strain. However, *M. smithii* was not consistently detected in all samples analyzed. Since the primers Probio_Uni and Probio_Rev match with a high score to the 16S rRNA gene sequences of this archaea, our results suggest incomplete lysis of archaeal cells. In this regard, *Methanobrevibacter* cells are covered by pseudomurein, a polymer that is similar to bacterial peptidoglycan with the exception of the l-N-acetyltalosaminuronic acid linked (β-1,3) to d-N-acetylglucosamine (GlcNAc) and the absence of D-amino acids in the interpeptide bridges [39]. Therefore, it seems that methods focused on pseudomurein digestion are needed for optimizing methanobacteria lysis. Overall, our results point that this microorganism is present in the human intestine in quantities higher than previously reported in some microbiota studies. Notably, our results indicate that several species, such as *F. prausnitzii, C. intestinalis* and *B. thetaiotaomicron,* are normally overestimated, whereas others, such as *B. longum*, are underestimated.

In order to determine which of the four tested DNA extraction methods provided the most reliable representation of the fecal bacterial community, we determined the proportion of each microbial group using the percentages of sequences obtained after 16S rRNA gene-based microbial profile analysis and compared this to the known number of microorganisms added to each sample. As previously shown, the evaluation of the abundance of members of the gut microbiota based on 16S rRNA gene profiling might be influenced by the copy number of 16S rRNA gene sequences [40].

Thus, we decided to normalize the number of reads linked to microbial taxon for the copy number of 16S rRNA gene loci present in their genome sequences (Table 2 and Figure S1). The detected difference, expressed in log units, between the number of microorganisms added and the number of sequences, either normalized for the copy number of 16S rRNA gene loci or not normalized, was calculated for each microorganism. The Bray-Curtis dissimilarity indexes were then calculated between the experimental data obtained for each bacterial mix (P1, P2 and P3) and the true levels of added microbes present in these mixes. This approach allows the identification of the best method without introducing subjective biases. In fact, the method showing the lowest mean value is considered to provide the most accurate overall representation of the bacterial communities in feces, based on the fact that the obtained Bray-Curtis dissimilarity index value is the lowest for the situation where the observed discrepancy between the number of microorganisms added and detected is the smallest. Thus, when normalization for the copy number of 16S rRNA gene loci was not considered, we found that the Qia-DNA extraction method yielded the most reliable results out of the four methods tested here, since it provides the most accurate reflection of the fecal microbiota composition. In contrast, the MBioEz-DNA extraction method generated the least accurate results. The Bray-Curtis distances using the four different methods were 0.17 (Qia), 0.23 (QiaEz), 0.29 (MBio) and 0.45 (MBioEz). However, if normalization for the number of 16S rRNA gene copies was taken into account, the Qia and QiaEz-DNA extraction methods showed the best results, with Bray-Curtis distance values being 0.17, 0.16, 0.39, and 0.42 for the Qia, QiaEz, MBio, and MBioEz-DNA extraction methods, respectively. We also noticed that an initial enzymatic treatment to enhance cell lysis increases the representation of some species, such as *B. thetaiotaomicron.* It is worthwhile mentioning that results are expected to be different when alternative DNA extraction protocols are used. In fact, other studies have recently indicated that the Qia-DNA extraction method is not as accurate as alternative bead-beating methods [41], [42].

**Table 2:** Relation between the percentages of 16S rRNA gene sequences obtained using the four different DNA extraction methods and the mixes P1, P2 and P3.

MIX	Species	Cellnumber	% eachstrain	16S copynumber	relative abundance not normalized				relative abundance normalized			
P1					MBio	MBioEz	Qia	QiaEz	MBio	MBioEz	Qia	QiaEz
	*B. longum*	3,84E+09	10,71	4	2,99	57,36	3,39	23,47	1,07	43,68	1,35	20,54
	*B. coccoides+B. producta*	7,68E+09	21,42	1+	1,61	1	3,5	0,58	2,30	3,05	5,59	2,03
	*C. intestinalis*	3,84E+09	10,71	2*	31,58	18,47	18,75	17,58	22,57	28,13	14,98	30,78
	*F. prausnitzii*	3,84E+09	10,71	1	47,14	4,62	42,04	3,61	67,38	14,07	67,19	12,64
	*E. coli*	3,84E+09	10,71	7	2,32	1,53	6,6	24,4	0,47	0,67	1,51	12,21
	*K. pneumoniae*	3,84E+09	10,71	8	3,45	2,6	11,37	4,18	0,62	0,99	2,27	1,83
	*P. copri*	3,84E+09	10,71	1*	2,23	0,41	2,09	0,58	3,19	1,25	3,34	2,03
	*B. thetaiotaomicron*	3,84E+09	10,71	5	8,38	13,2	11,74	25,47	2,40	8,04	3,75	17,84
	*M. smithii*	1,30E+09	3,63	2	0,01	0,08	0	0,06	0,01	0,12	0,00	0,11
**P2**												
	*B. longum*	3,84E+09	18,59	4	5,64	18,59	3,52	2,15	1,72	7,21	1,11	0,69
	*B. coccoides+B. producta*	7,68E+09	37,18	1+	2,86	2,43	9,06	6,35	3,49	3,77	11,42	8,17
	*C. intestinalis*	3,84E+09	18,59	2*	27,11	33,14	32,12	35,76	16,56	25,72	20,25	23,01
	*F. prausnitzii*	3,84E+09	18,59	1	63,94	39,88	52,87	52,33	78,12	61,90	66,66	67,35
	*E. coli*	3,84E+07	0,19	7	0,08	3,21	0,63	0,8	0,01	0,71	0,11	0,15
	*K. pneumoniae*	3,84E+07	0,19	8	0,05	1,26	0,82	1,2	0,01	0,24	0,13	0,19
	*P. copri*	3,84E+07	0,19	1*	0,03	0,12	0,1	0,17	0,04	0,19	0,13	0,22
	*B. thetaiotaomicron*	3,84E+07	0,19	5	0,19	0,84	0,75	0,86	0,05	0,26	0,19	0,22
	*M. smithii*	1,30E+09	6,29	2	0	0	0	0	0,00	0,00	0,00	0,00
**P3**												
	*B. longum*	3,84E+07	0,23	4	0,1	0,2	0,05	0,29	0,09	0,26	0,05	0,42
	*B. coccoides+B. producta*	7,68E+07	0,46	1+	0	0	0,02	0,02	0,00	0,00	0,09	0,12
	*C. intestinalis*	3,84E+07	0,23	2*	0,32	0,33	0,14	0,2	0,57	0,86	0,31	0,58
	*F. prausnitzii*	3,84E+07	0,23	1	0,35	0	0,29	0,08	1,24	0,00	1,27	0,47
	*E. coli*	3,84E+09	22,79	7	14,06	14,33	24,5	48,13	7,10	10,67	15,28	40,15
	*K. pneumoniae*	3,84E+09	22,79	8	27,77	17,97	36,86	9,24	12,28	11,70	20,12	6,74
	*P. copri*	3,84E+09	22,79	1*	13,86	1,53	8,75	0,48	49,01	7,97	38,21	2,80
	*B. thetaiotaomicron*	3,84E+09	22,79	5	42,02	65,4	28,26	41,46	29,72	68,15	24,68	48,42
	*M. smithii*	1,30E+09	7,71	2	0	0,15	0	0,1	0,00	0,39	0,00	0,29

The sequence of *B. coccoides* and *B. producta* were indistinguishable and were included in the same group.

* Means that only draft genomes are available, thus the 16S copy number is probably underestimated.

+ Means that no sequenced genomes are available, thus the *genera* average 16S copy number was used.

The normalized relative abundance was calculated by division of the relative abundances of every species by their predicted 16S copy number. Results of each sample were normalized so that their sum is 100%.

### Comparison of Gut Microbiota Profiling using Different Primer Sets

**Figure 3:**
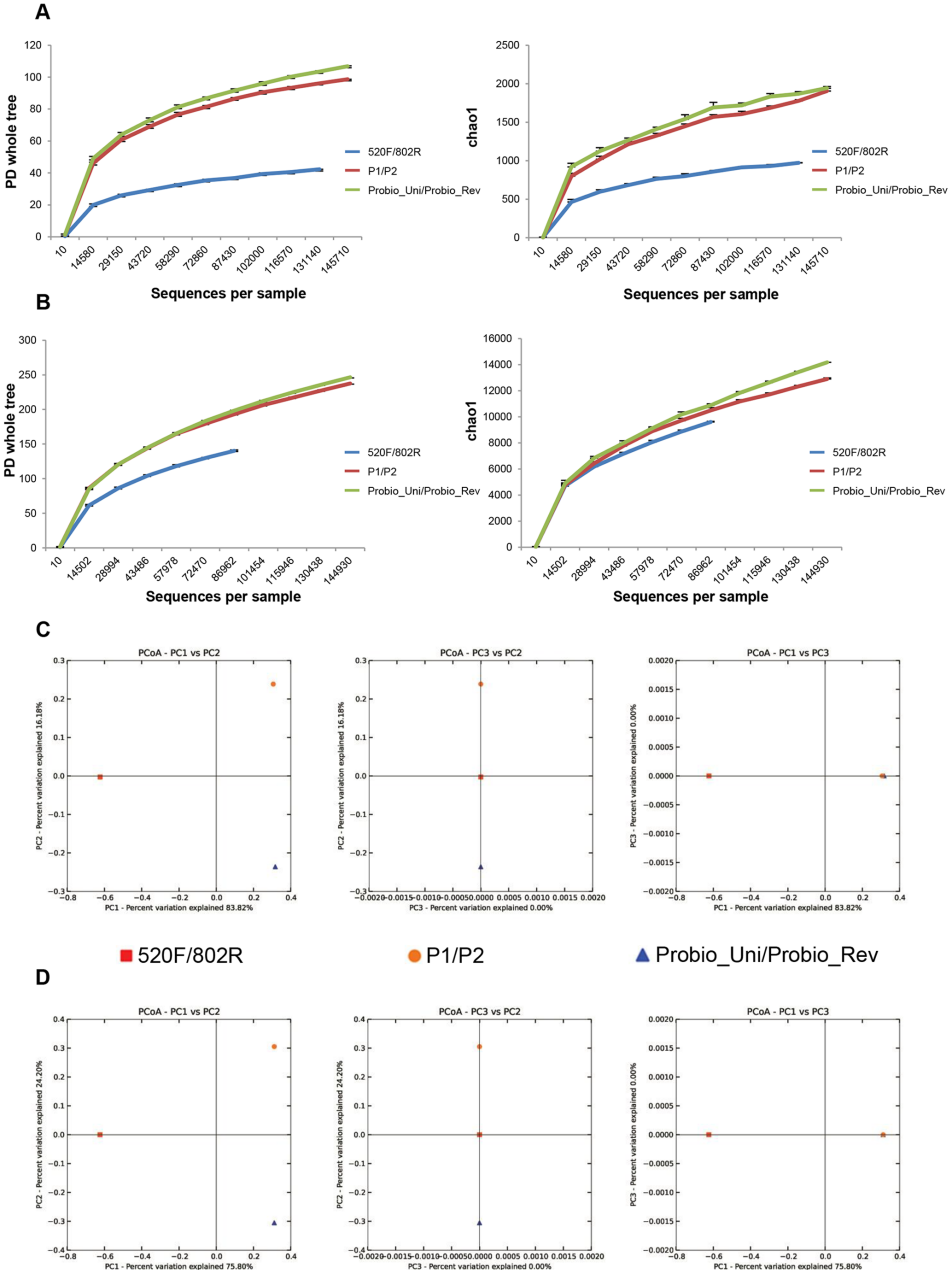
Rarefaction curves generated for 16S rRNA gene sequences and Principal Coordinate Analysis (PCoA) based on the phylotypes identified from different PCR primer sets as well as from different samples (stool samples of infants and fecal samples of mothers). Panels a and c display the rarefaction curves and the PCoA from stool samples of infants. Panels b and d show the rarefaction curves and the PCoA from fecal samples of mothers. In panels c and d percentages shown along the axes represent the proportion of dissimilarities captured by the axes. Each symbol represents the 16S rRNA gene sequences from each sample which are displayed in a different colour and shape according to the PCR primer pair. In panels a and b the plots depicted on the left represent the rarefaction curves determined using the PD index whereas the plots show on the right constitute the rarefaction curves obtained using the Chao index. A 95% confidence intervals was added to the rarefaction curves.

In order to further evaluate the efficacy of different primer sets, including Probio_Uni/Probio_Rev, P1/P2 [27], 520F/802R [43] to delineate the microbiota composition of human fecal samples, we sequenced 16S rRNA gene-based amplicons achieved with either of these primers pairs, using the same DNA stock extracted from two different fecal samples. For this purpose two human fecal DNA samples were used, one retrieved from a three month infant stool sample, which based on previous published data was considered to possess a low level of complexity [18] and another one extracted from a mother’s fecal sample assumed to include a diverse composition of microbiota [18], [32], [43]. In total, 1,276,969 sequence reads, representing 633,877 and 643,092 reads per infant- and mother sample, respectively, were generated on the Ion Torrent PGM machine (Table 3). The decrease in the rate of phylotype detection and the plateauing of various diversity indices for this set of PCR primers demonstrated that a large part of the diversity in these libraries had been detected, even though a higher number of phylotypes was identified for the primer pair Probio_Uni/Probio_Rev as compared to the other PCR primer sets (Fig. 3).

**Table 3:** Quantitative data of the 16S rRNA gene sequence datasets used in this study.

Dataset*	Sample	Numberof reads	Number of reads removed because of:						Final read number	Reduced by (%)
			Outside bounds (160–300)	Ambiguous bases	Mean quality<15	Homopolymer runs >6bp	Primermismatch >1	Low quality window truncation results in <160bp		
**M**	M520F/802R	171559							103757	39.52%
	MProbio_Uni/Probio_Rev	231660							146688	36.68%
	MP1/P2	239873							152995	36.22%
	**TOTAL**	**643092**	**130387**	**0**	**0**	**18290**	**25459**	**65516**	**403440**	**37.27%**
**I**	I520F/802R	180917							133592	26.16%
	IProbio_Uni/Probio_Rev	251632							151790	39.68%
	IP1/P2	186780							145730	21.98%
	**TOTAL**	**633877**	**37396**	**0**	**8060**	**6029**	**90701**	**59878**	**431112**	**31.99%**

* The M dataset stands for mother’s fecal sample dataset; I dataset correspond to the infant’s stool sample dataset.

The significance test in UniFrac [44] reported P-Values (Bonferroni corrected) equal to 1e-02 for every pair of samples of both mother and infant sets, meaning that the results are highly significant (if P-values ≤0.05 are considered to be statistical significant). This method was used to evaluate if the cluster distribution of the sequences achieved with the different PCR primers differs from random expectations. Principal Coordinate Analysis (PCoA), applied using the UniFrac program, showed that, while the datasets obtained with the P1/P2 and Probio_uni/Probio_rev primer pairs cluster together, the dataset achieved with the 520F/802R primer pair is rather different (Fig. 3). This suggests that the microbial composition assessed by this latter primer set is different than those detected by either P1/P2 or Probio_uni/Probio_rev.

**Figure 4:**
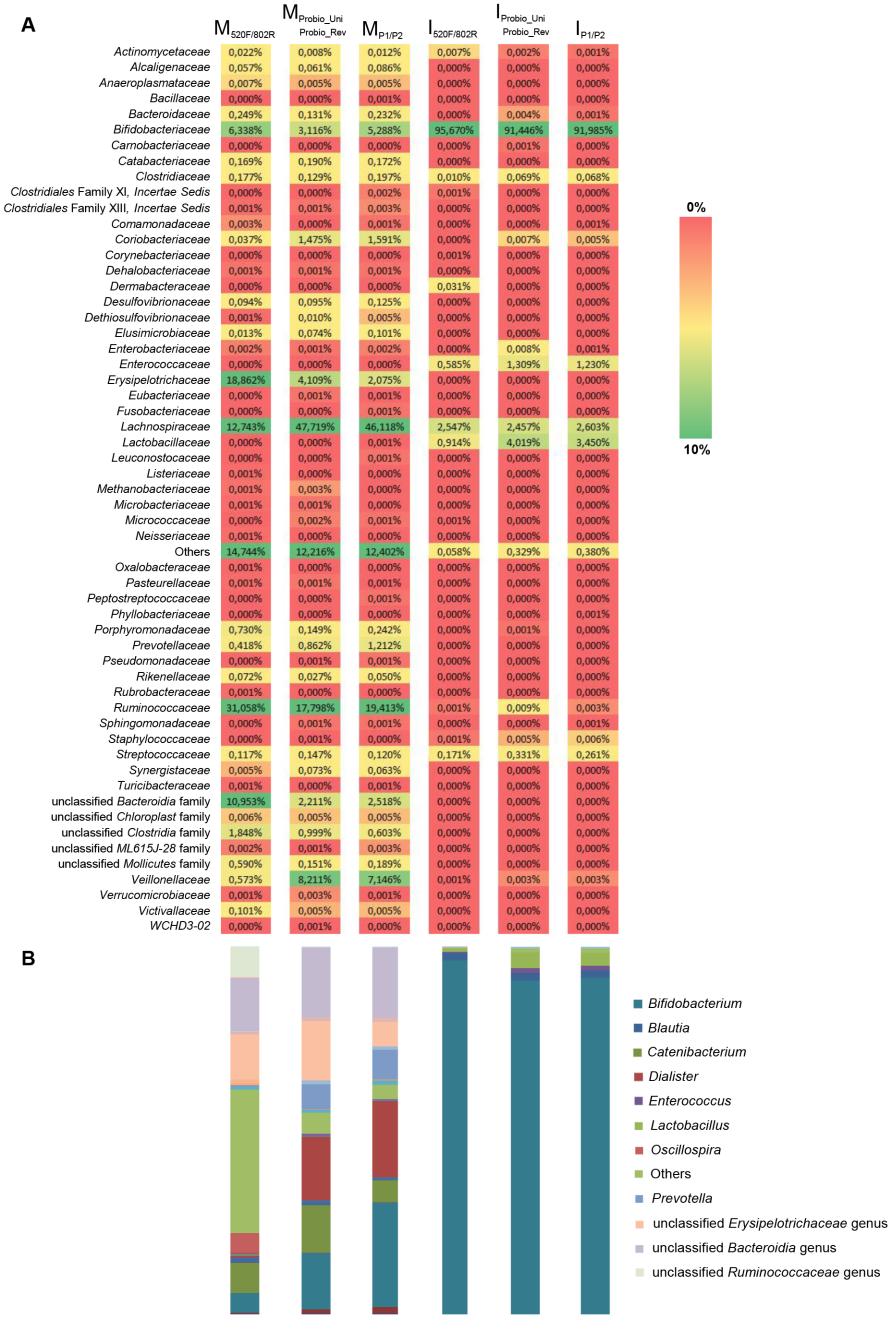
Number of sequences per phylotype for each PCR primer pair and sample (stool samples of infants and fecal samples of mothers). The y axis displays the OTUs at family level detected in this study; each row represents a different OTU. Increasing darkness of the colour scale corresponds to higher estimated relative abundance. At the bottom of the heatmap a representation of the abundance of different phylotypes at the genus level detected by different PCR primer set for each sample has been displayed.

Clustering of de-noised high quality reads generated 18,910 and 2,361 OTUs for the mother and infant samples, respectively. As expected the microbial composition of the two fecal samples displayed a much simpler OTU organization in the infant stool sample as compared to that of the mother. At genus level, sequencing reads could be assigned to 83 main individual taxons, of which 30 were present in all the three PCR-primer dataset in at least one of the two samples (Fig. 4). Furthermore, we analyzed the similarities and differences in the composition of the fecal sample assayed using the three PCR primer sets by hierarchical clustering based on population profiles of the most common and abundant taxa (Fig. 4). The clustering patterns are also reflected in the corresponding bar diagram (Fig. 4), highlighting bias in resolution of the PCR primer sets against specific microbial taxa. Microbiota composition observed with these different primer sets, highlighted a discrepancy with respect the *Enterococcaceae*, *Lachnospiraceae*, *Lactobacillaceae*, *Streptococcaceae* and *Veillonellaceae* (Fig. 4). In fact, primer set Probio_Uni/Probio_Rev displayed a higher proportion of members of these bacterial groups compared to the other set of primers, where a lower number or no phylotypes corresponding to the above mentioned microbial taxa were identified (Fig. 4). The latter finding thus reinforces the importance of a reliable PCR primer set for the appropriate delineation of the biodiversity of the human gut.

### Conclusions

Appropriate primer selection as well as DNA extraction protocols in microbiota studies using 16S rRNA gene sequencing approach is essential to enable trustworthy representation of the organisms present in an environment such as human gut ecosystem. In this context, dominant infant gut microbiota members such as bifidobacteria are under-represented in many published metagenomic studies of the microbial biodiversity of the infant gut [22] due to technical biases. In fact, the presence of a thick cell wall of bifidobacterial cells as well as a polysaccharide surface layer [45] might render these microorganisms recalcitrant to cell lysis, thus causing a low recovery of their chromosomal DNA. In contrast, other key members of the gut microbiota such as *Faecalibacterium* and *Bacteroides* might be over-represented in most of the current human gut metagenomic studies. Here, we were able to demonstrate that the bias observed against the detection of bifidobacteria was due to the DNA extraction as well as PCR steps. We have shown that erroneous conclusions about the presence/absence as well as relative proportion of bifidobacteria are likely if primers that do not sufficiently complement the target 16S rRNA gene sequence are used. Noticeably, the error frequencies predicted to occur within DNA sequences generated by IonTorrent is equivalent to that estimated for other NGS technologies such as 454, i.e., about 1% [9]. Such a low level of error should not affect the final OTUs prediction, since these are calculated at 97% of nucleotide identity.

In this study we have designed a PCR primer set allowing accurate detection of bifidobacteria as well as other main members of the human gut microbiota, which, in combination with a fast and cheap sequencing approach like the Ion Torrent PGM, is suitable for the investigation of the microbial composition of the human gut microbiota.

## Supporting Information

Figure S1 **Ratio of 16S rRNA gene sequences obtained after the analysis of the artificially contaminated gnotobiotic fecal samples.** Four different DNA extraction procedures (Qia, QiaEz, MBio and MBioEz DNA-extractions) and three different mixes of microorganisms (P1, P2 and P3) were used (see material and methods section). A, B and C represent the results obtained for the three different mixes (P1, P2 and P3, respectively) when the relative abundance of 16S rRNA gene sequences was not normalized. D, E and F represent the results obtained for the three different mixes (P1, P2 and P3, respectively) when the relative abundance of 16S sequences was normalized considering the predicted 16S copy number of the strains. In the center of each panel, the expected result according to the real microbial population present in each sample is depicted, and the graphics on the corners show the results using the four different extraction methods. The sequence of *Blautia coccoides* and *Blautia producta* were indistinguishable and were included in the same group.
(TIF)
