# Supplementary figures and images for: Assessing the Fecal Microbiota: An Optimized Ion Torrent 16S rRNA Gene-Based Analysis Protocol
Source: PLoS One. 2013 Jul 15;8(7):e68739. doi: 10.1371/journal.pone.0068739 (PMC3711900; doi:10.1371/journal.pone.0068739)

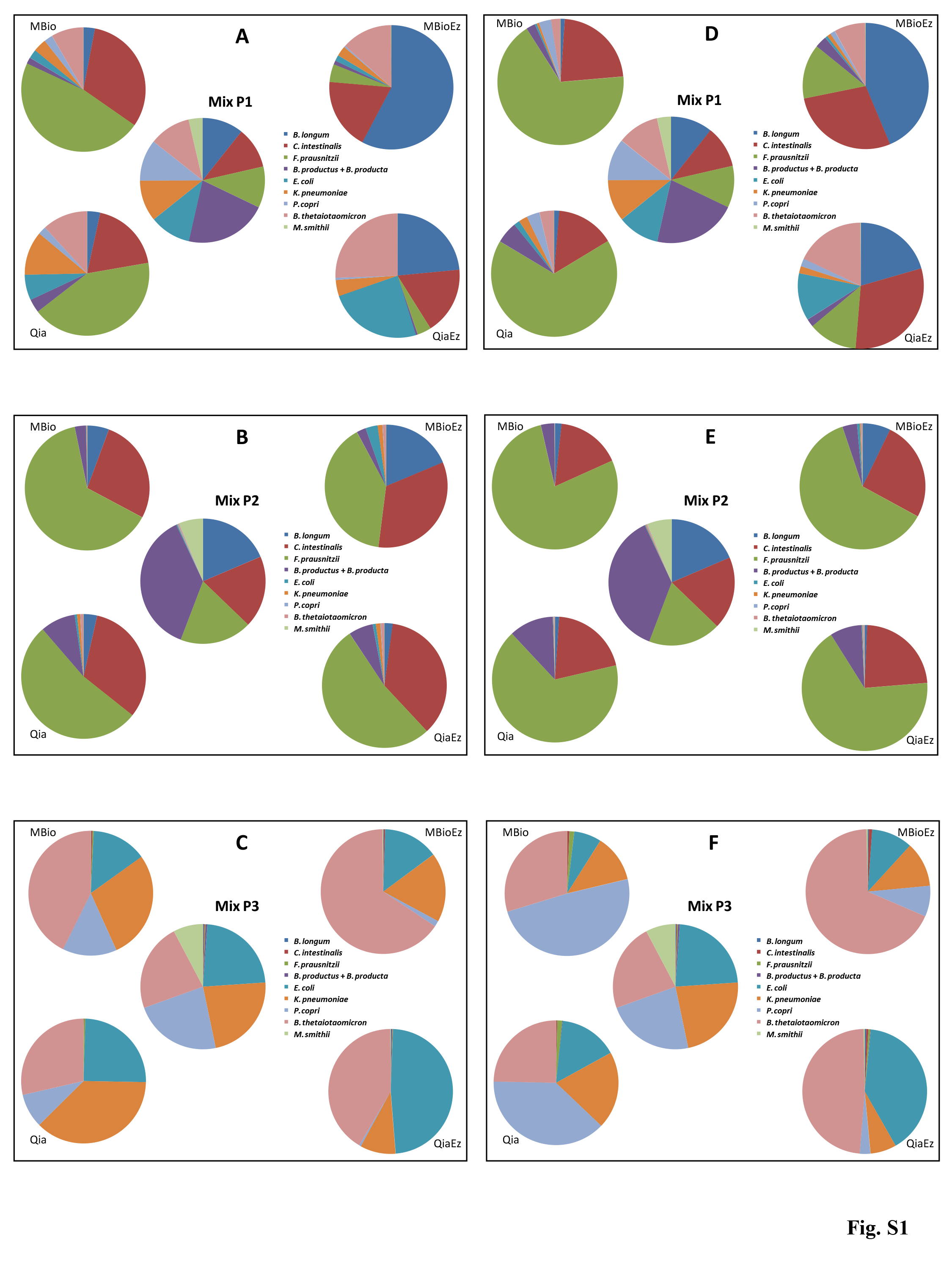

Supplement: Figure S1 — Ratio of 16S rRNA gene sequences obtained after the analysis of the artificially contaminated gnotobiotic fecal samples. Four different DNA extraction procedures (Qia, QiaEz, MBio and MBioEz DNA-extractions) and three different mixes of microorganisms (P1, P2 and P3) were used (see material and methods section). A, B and C represent the results obtained for the three different mixes (P1, P2 and P3, respectively) when the relative abundance of 16S rRNA gene sequences was not normalized. D, E and F represent the results obtained for the three different mixes (P1, P2 and P3, respectively) when the relative abundance of 16S sequences was normalized considering the predicted 16S copy number of the strains. In the center of each panel, the expected result according to the real microbial population present in each sample is depicted, and the graphics on the corners show the results using the four different extraction methods. The sequence of Blautia coccoides and Blautia producta were indistinguishable and were included in the same group. (TIF) [file pone.0068739.s001.tif]
